# Routine Practice

**Published:** 1883-10

**Authors:** P. P. Wells

**Affiliations:** Brooklyn


### ROUTINE PRACTICE.

P. P. Wells, M. D., Brooklyn.

It was in our way to observe, not long since, on hypothesis as an impediment to progress in professional knowledge. We still think it one of our greatest hinderances, and are inclined to place next to it in importance that resort of the ignorant and idle—routine. It is so easy to do to-day what we did yesterday for difficulties which we too readily assume to be identical with those called by the same name which we treated yesterday. This is so much easier than the thorough investigation of the new case, which alone can bring its true nature and relationship to curatives into light, and show the innate differences which exist in cases called, by reason of our poverty of nomenclature, by the same name. But ignorance and idleness are not alone responsible for this great practical evil. It has its origin largely in a radical defect in our practical methods in dealing with the sick—a defect which present teaching and current thought are just now greatly extending and intensifying. Indeed, it may be said that this defect has become a fashion, and the doctor who has not come under its control is almost as much of a spectacle in practice as our excellent grandmothers would be socially if they were to appear in the garb which half a century ago was thought so very nice and becoming. The fashion to which we allude is that of attempting the practice of Homœopathy from an allopathic standpoint, which is about as wise as would be an attempt to mix light and darkness. The objectives of the two schools, at the beginning of practical dealing with sickness, have nothing in common. "What is your first thought in your treatment of sickness?" was an inquiry of one of our school well known to every reader of this paragraph, addressed to an old-school practitioner of some eminence. "To make my diagnosis," was the reply. "That is, *to give a name.* I thought so," was the answer of our friend. "And pray, what may be your first thought?" was the rejoinder of the old-school doctor. "To find what will *cure* my patient," was the reply. The *name* on the one side, and the *curing agent* on the other, and here in a nut-shell you have the characteristics of the two schools as given by two representative practitioners when each questioned the other. The *name,* and treat that according to tradition on the one side; *the totality of the symptoms disclosing the curative* on the other—and *voila* old-school physic and Homœopathy in briefest terms.

Now, in view of this contrast, what reasonable inducement can there be for the prescriber of specific medicine to run wildly and madly after that which is only, at the best, shadowy, and not infrequently false, leaving, in so doing, the only facts which, under the law, will guide him to a speedy and comparatively a certain cure? Why leave facts and law for a name? And yet, unless we mistake the signs of the times, toward this the teachings and the fashion of practice of the present strongly tend. And more than this, it is apparently carrying with it the general popular approval. Make your diagnosis, *i. e.*, find the symptoms which justify the name, and then—name being *a*, is it not accepted that *drug x* cures *disease a?* and what is *easier* than to give *x?* and there is an end of all trouble. *Ease* and one other fact, and beyond these we can see no possible excuse reasonable men can shield themselves behind for this departure from the spirit and teachings of our divine law. Indeed, it is an utter abandonment of all which is characteristic of its teaching and guidance in finding the specific curative for our case, which alone constitutes any practice homœopathic.

The other fact is the charm for so many which seems to lurk in the words, "*scientific medicine!*" Is not the "scientific" the opposite of ignorance? And does not the old school, and constantly, call itself "scientific medicine"? Would there be so great persistency in this if there were no foundation for the claim it thus sets up? And why not come as completely under the shadow of this pretense as is possible, and still retain our name and character of homœopathic practitioners? We answer, because there can be no mingling of truth and falsehood. There is no truth but only falsehood in this claim so set up and insisted on, and to imitate this practice, founded on *name*, the most unscientific conceivable, or to attempt this, is to abandon that in which alone exists any "science of therapeutics," and go into the practical outer and utter darkness which envelops the old school, from which the revelations of the new have so happily delivered us. And yet the attempt to do this constitutes the fashion and the folly of routine so characteristic of the prescribing of the present time. For the same *name* give the same *drug* or drugs. This is the true worship of the great image which modern Nebuchadnezzars of the old school have set up—diagnosis—and called it "*scientific medicine*."

This fault is not in diagnosis; it is always well to have this rightly made, and there is nothing to be said in disparagement of it. The fault is in putting this as the basis of therapeutics, instead of the law which constitutes the only true foundation of this science. With the old school, this is natural and plausible enough, as they have no law to guide them in their attempts to apply their therapeutics for curing the sick; with the homœopathists, who have, to abandon this sure guide and go after this shadow of false pretense is no better than a crime.

Now, Homœopathy rejects both ease and this false pretense of diagnosis as a basis of therapeutics, and all other temptations to routine, and only accepts a practice based on the *totality of the symptoms* of the case to be treated; it insists on all, however difficult of attainment or however numerous they may be. This it does for this reason: Till the *whole* sum of them is known, that which is most authoritative in the choice of the remedy may be wanting, and therefore the wrong drug may be selected; and this is always an embarrassment in subsequent treatment of the case and not unfrequently the cause of its fatal termination. This is the more conspicuously true in those forms of disease which run a violent and brief course with rapid tendency to dissolution. Of this class scarlet fever is one of the most important; and to this may be added diphtheria. The first prescription in severe cases must be right, or subsequent ones may be of very little consequence. There may be no time or opportunity to correct mistakes if made here. We give the following cases of this fever, on which we propose to make a few remarks where they illustrate or throw light on this matter of routine:

H. W. E., 7 years old, went to the breakfast table the morning of June 22d, 1883, and refused all food except a banana. He ate one and left for his room. He almost immediately vomited what he had eaten and said he wanted to go to bed. The vomiting was immediately followed by violent headache; flushed and congested face, eyes, and external head; this was hot and dry, as was the skin of the whole body; the pulse 140, but moderately developed; the throat became inflamed; swallowing very painful; he was restless and at the same time drowsy. There was no difficulty in seeing scarlet fever, though the most careful examination failed to find any trace of its eruption, which in attacks so sudden and violent as this usually appears early. The symptoms were more like *Belladonna* than any other recognized drug, and he got the 200th potency of it in water, a teaspoonful every three hours.

The morning of the 23d found him relieved of his headache and congestion of the face and eyes, but the throat, pulse, and general symptoms were the same as the day before. The pain when swallowing passed from the throat to the ear. The throat affection was worse on the right side. He got Lachesis 200 at 8 o'clock A. M. At 7 P. M. the throat and other symptoms were no better. After a careful revision of the case he got *Merc. sol.* 200 in water, a teaspoonful every three hours.

24th, 8 A. M.—When asked how he was, he answered, in a bright, cheerful voice, "All right." He had now no pain of any kind and swallowed without inconvenience. The pulse was still 140, with entire absence of appetite. There was still no sign of eruption. He got *Lachesis* 200.

25th, 8 A. M.—The rash was fully out but there was no diminution of the frequency of the pulse or fever. He continued in this state until the fourth day of the rash, when this began to fade, the pulse remaining as before. This led to inquiries as to the renal functions, and it was found he was passing less than a half pint of water in twenty-four hours of dark, brownish-colored urine, and this was passed but once, or at most twice, in this time. After a careful review of this case he got *Cantharis* 200 in water, a teaspoonful every three hours.

June 30th.—Urine much increased in quantity but of the same character, with a brown sediment of fine, granular appearance. Pulse the same as before. No appearance of desquamation.

July 1st.—He got one dose of *Sulph.* 200 dry, and the next day desquamation appeared on the limbs. No change in the urine or pulse. The Sulphur was left to do its work till

July 5th—When, in view of the character of the urine, the pulse, the dry skin, the slow progress of the desquamation, he got *Lachesis* 200, with no perceptible result.

July 7th.—He got *Ambra* 200.

July 8th.—The *color* of the water natural; the sediment, pulse, and skin unchanged. Ambra was continued without further benefit till

July 12th—When, as Lachesis seemed to correspond to his symptoms better than any other drug, and notwithstanding this, in the 200th potency, given on the 5th, had produced no beneficial result, it was decided to give it again and *higher*. A few pellets of the 5mm of Fincke were dissolved in half a goblet of water and a teaspoonful given at 8 o'clock A. M.

July 13th.—No sediment in the urine. Its color and quantity were normal. Pulse, 80. From this time convalescence was progressive till it was complete. He got no more medicine.

There could be no doubt as to the first prescription. It could be no other than *Belladonna*. It seemed to touch all points of the case and was given for this reason, not because it was once accepted as the specific for this fever. The result was a little curious. It removed one group of symptoms perfectly, not to return again in the history of the case, while it left all others untouched. It is not unreasonable to suppose that it effected more than this, in securing for the little patient his perfect immunity from pain * (except that in the throat during deglutition) through his subsequent confinement. But why did this remedy leave the throat troubles so entirely unaffected when it has so potent relations to the affections of this region? It was a disappointment that it should do this in this case, as, in addition to the pain and congestion of the head, etc., it seemed to promise relief here also by its general likeness to the local phenomena here present. The result was a demonstration of a truth we knew before—that the curative relationship between drugs and sickness is not in the *general*, but in the *specific*, phenomena of the two. Hence, the imperative duty of most thorough and minute examination of cases before deciding the question of the prescription.

---

* This absence of pain was rather forcibly and whimsically expressed by the little fellow in an attempt he made, the second day after the relief of his throat troubles, to write a letter to his next-door playfellow, then absent in the country. He began his letter thus—" Dear Stanley, *scarlet fever does not hurt a bit.*"

Then the answer to Why did not Lachesis relieve the throat? is not unlike the above. It has, besides its likeness to the trouble to be removed, a certain relationship, or is supposed to have, to the scarlet condition, and yet it failed. The trouble was perfectly and permanently removed by Mercury, which, if it has any such relationship, has it in a less degree than its near relative. The cure in a given case does not in the least depend on the chosen remedy having been given many times and unsuccessfully in treating other cases, called by the same name, but solely on the likeness of the specific phenomena of remedy and disease. This and one other lesson perhaps, which we shall call to mind by and by, is taught by this failure of Lachesis.

Then, as to the struggle for the protection of the kidneys: Why did not Lachesis, previously given, remove the threat of destruction, or, rather, why not prevent it? Was it that Lachesis is marked in its primary effects by increased urinary secretion, while here it was so notably diminished? It may be. The remedy first given was selected after mature consideration, though I have no knowledge of its having been given before in any case of scarlet fever, or of its having ever been supposed to have curative relationship to it. It restored the *quantity* of the secretion all the same and as perfectly as if it had had the authority of use for generations. This was regarded as an important gain, though, as, with other remedies, it meddled only with its own symptoms, leaving all others as it found them.

Lachesis 200 failed again, July 5th, to change the color or to remove the sediment of the urine, though it seemed to fulfill the conditions of the law in the case, and the patient got, after this failure, Ambra gris., which restored the normal color of the secretion, but left the sediment as before—dark brown, and apparently composed of minute granules. After waiting five days for a change in this symptom and finding none, and after a protracted study for a remedy more like to this and all the other remaining phenomena of the case, the choice again fell on Lachesis, though the little fellow told his prescriber he "did not think he was a Lachesis boy," and notwithstanding the repeated disappointments which had followed its use. In these circumstances it was determined to give the remedy *higher*. The only higher number than 200 in possession of the prescriber was very high—so much so as, perhaps, to be regarded generally rather as a curiosity than as a remedy for practical use. In the present case, however, it was not regarded altogether as a curiosity, for when, as an experiment, it had been given to a young lady suffering from a peculiar chronic throat affection which had been for a long time a trouble, this was promptly, permanently, and, we may add, unexpectedly cured. We dissolved a few minute pellets of Fincke's 5mm of Lachesis in half a goblet of water, and gave the little patient one teaspoonful. In twelve hours from the taking of this dose there were no more symptoms to treat. What would have been the effect if we had given this number the first time we gave the lower 200? We can never feel quite sure that the whole case would not have been wiped out at the first, as the whole remainder was when now it was given in this late stage of the case. Here is the second lesson to which we alluded a moment ago: The *right* potency is not an unimportant matter. Was it such prescriptions as this to which our late President of the American Institute alluded when he called them "*extreme*," and the prescribers "extremists," by this seeming to hold both up to the contempt of members? The perfection of this cure of this obstinate case was certainly "*extreme*." When will our presidents in their addresses cease to be silly?

As an addendum to the above case, and further to illustrate the true character of *homœopathic* prescribing and its superiority over that based on diagnosis, or, what is the same, on a *name*, we give the following case, the patient being the mother of the little patient whose case we have just considered. It may not be amiss to say she, and all the members of the family, exposed to contagion by this case, took Bell. 200, morning and evening, for the ten days next following his attack, and that, excepting the mother, no one of them, six in number, was affected in the least by a continued residence under the same roof through the fever and its convalescence.

On the 5th day after beginning of desquamation, in the preceding case, the mother, who had been his constant attendant, day and night, from the first, was suddenly attacked with pain in the throat, increased greatly by every attempt to swallow; great sensitiveness to touch on the outside and right of the neck; and curious enough it was, that when she swallowed it was in this outside locality that the pain was greatest. The pulse was quick, 120 in the minute; skin hot and dry; general sense of malaise great; face flushed; eyes injected. In short, it seemed an unmistakable case of the same trouble her boy was just passing through. No doubt every diagnostician, old school and new, would have called it by the *same*

*name.* And every routinist of the new would have been tempted by the congestion of face and eyes, the sore throat, the protracted exposure to the scarlet poison, to give the supposed specific for scarlet fever—*Belladonna.* We say the routinist would have been so tempted; the true prescriber would not. For, though there were several symptoms in the case very like to those of Bell., notably the congestion of face, pulse, painful swallowing, etc., there were others, and those the most important therapeutically, not found in the pathogensis of this drug. Especially we should in it seek in vain for *pain in the right outside of the neck when swallowing, and for the extreme sensitiveness of this part to touch.* These were the "*peculiar symptoms*" of the case, which the *Organon of Homœopathic Medicine* says [Sec. 153, page 173 *et seq.*] are to be "chiefly regarded" in the choice of the curative, and were at once recognized as the characteristic symptoms of the case for which the most similar remedy was to be found before any prescription in the case could be homœopathic. These symptoms are not found in any of the remedies most frequently in use in the treatment of this fever. What then shall be done? Shall we select from these the one most frequently given in cases of this fever (routine), or shall we seek a simillimum of these "peculiar symptoms," *i. e.*, really proceed with the case homœopathically? We decided on this latter course, and found the likeness of these uncommon symptoms in a drug of very uncommon use. Indeed, it was found in a member of the materia medica not called for in any one case in the prescriber's more than forty years of seeking specifics for the cure of the sick, and when found to be most like and very like the whole group of symptoms of the case, these "peculiar symptoms" included, *Nickel* was given with the utmost confidence, and the result justified the confidence. Every trace of scarlet fever affection was gone in less than twelve hours.

Continuing in the atmosphere of this poisonous exfoliation, this mother was attacked a second time with similar symptoms, just one week after the disappearance of those of her first attack. She had a second dose of the same remedy, and this attack yielded as promptly as the first and the cure was permanent, though she still continued her cares as nurse of her boy.

What had diagnosis to do with this cure? Is the proper function of the doctor to *cure*, or *to give names?* Who will say this prescription was not "scientific medicine"?

